# Topical and Systemic Therapeutic Approaches in the Treatment of Oral Herpes Simplex Virus Infection: A Systematic Review

**DOI:** 10.3390/ijms26178490

**Published:** 2025-09-01

**Authors:** Antonio Mancini, Angelo Michele Inchingolo, Grazia Marinelli, Irma Trilli, Roberta Sardano, Carmela Pezzolla, Francesco Inchingolo, Andrea Palermo, Gianna Dipalma, Alessio Danilo Inchingolo

**Affiliations:** 1 Department of Interdisciplinary Medicine, University of Bari “Aldo Moro”, 70121 Bari, Italy; dr.antonio.mancini@gmail.com (A.M.); angelomichele.inchingolo@uniba.it (A.M.I.); graziamarinelli@live.it (G.M.); trilliirma@gmail.com (I.T.); roberta.sardano@uniba.it (R.S.); carmela.pezzolla@uniba.it (C.P.); gianna.dipalma@uniba.it (G.D.); alessiodanilo.inchingolo@uniba.it (A.D.I.); 2 Department of Biomedical, Surgical and Dental Sciences, Milan University, 20122 Milan, Italy; 3 Department of Experimental Medicine, University of Salento, 73100 Lecce, Italy; andrea.palermo@unisalento.it

**Keywords:** oral herpes simplex virus, HSV-1 infection, herpes labialis, recurrent herpes, antiviral therapy, topical treatment, systemic treatment, viral latency, viral reactivation, therapeutic strategies

## Abstract

Herpes Simplex Virus (HSV) infections, caused primarily by HSV-1 and HSV-2, are among the most prevalent viral diseases worldwide, with recurrent manifestations that significantly affect quality of life. Therapeutic strategies include both topical and systemic interventions, each with distinct goals. This systematic review was conducted according to PRISMA guidelines. A comprehensive search of PubMed, Scopus, and Web of Science (2005–2025) identified studies evaluating topical or systemic treatments for HSV. Eligible studies included randomized controlled trials and observational studies reporting validated clinical outcomes. Topical treatments, including acyclovir cream, docosanol, and newer formulations, primarily reduce lesion duration and alleviate local symptoms when applied early. These interventions have limited systemic absorption and generally do not influence recurrence frequency. Novel delivery methods and combination strategies, such as acyclovir–hydrocortisone formulations or photodynamic therapy, may enhance local efficacy and symptom control. Systemic Therapies: Systemic antivirals, such as acyclovir, valacyclovir, and famciclovir, target both lesion resolution and recurrence prevention. Evidence from randomized trials supports their use for episodic and suppressive therapy, including short-course, high-dose regimens that improve adherence while controlling symptoms. Systemic therapy is particularly indicated for recurrent, disseminated, or high-risk infections. Topical and systemic therapies serve complementary roles in HSV management. Topical agents are useful for localized or initial episodes, while systemic therapy addresses broader clinical objectives, including recurrence reduction. Future research should focus on mechanism-based therapies, novel delivery systems, and standardized outcome measures to guide personalized treatment strategies. Emerging therapies targeting viral latency, immune modulation, and gene-editing technologies hold promise for long-term suppression and personalized management of HSV infections.

## 1. Introduction

### 1.1. Therapeutic Strategies and Resistance in HSV

Herpes Simplex Virus (HSV), comprising two types, HSV-1 and HSV-2, is a widespread human pathogen responsible for recurrent infections, such as orolabial and genital herpes [1,2,3,4]. HSV-1 is typically associated with oral and ocular lesions, while HSV-2 is more commonly implicated in genital disease. Both types establish lifelong infections by entering a latent state in sensory neurons, particularly in the trigeminal and sacral ganglia. Periodically, the virus may reactivate, leading to recurrent symptomatic episodes or asymptomatic viral shedding [5,6,7]. Despite the chronic nature of HSV infections, therapeutic options remain limited. Systemic antivirals—including acyclovir, valacyclovir, and famciclovir—form the cornerstone of treatment, reducing symptom duration and viral shedding without eliminating latent virus. Topical treatments are primarily used for orolabial lesions, offering modest benefit when applied early during reactivation [8,9,10,11,12,13,14,15].

The persistence of HSV in a latent state and its ability to evade immune detection represent major barriers to achieving a definitive cure. In recent years, attention has shifted toward new therapeutic approaches that go beyond symptom management [16,17,18,19,20]. These include agents targeting the epigenetic regulation of viral latency, immunomodulatory therapies aimed at enhancing host defenses, and gene-editing technologies designed to eliminate latent viral genomes [21,22,23,24,25,26,27]. Recent research has focused on therapies that extend beyond symptom management, including agents targeting epigenetic regulation of viral latency, immunomodulatory approaches to enhance host defense, and gene-editing technologies designed to eliminate latent viral genomes.

This systematic review aims to provide an updated overview of both topical and systemic therapeutic strategies for HSV infections, with a particular focus on how advances in the understanding of viral latency and reactivation are shaping new clinical approaches.

### 1.2. Molecular Basis of HSV Latency and Therapeutic Implications

HSV latency is a critical determinant of therapeutic outcomes. After primary infection, the virus travels along sensory neurons to neuronal ganglia, where it establishes a dormant state. During latency, the viral genome persists as a circular episome, largely transcriptionally silent except for the latency-associated transcript (LAT), which helps maintain latency through apoptosis suppression, heterochromatin formation on lytic gene promoters, and modulation of host immune signaling. HSV latency is maintained by epigenetic silencing of lytic promoters through histone modifications (H3K9me3, H3K27me3), recruitment of host repressors (HDACs, polycomb proteins), and the activity of viral non-coding RNAs such as LAT, which prevents apoptosis and promotes heterochromatinization [28,29,30,31,32,33,34,35,36,37]. This molecular interplay has therapeutic implications, inspiring two main strategies: ‘shock and kill,’ which uses HDACi or EZH2 inhibitors to force viral reactivation, and ‘block and lock,’ which aims to strengthen heterochromatin and prevent viral gene expression [38]. Agents targeting lysine methyltransferases or bromodomain-containing proteins are under investigation, with attention to preserving normal host epigenetic function [39,40,41,42]. Recent preclinical work has confirmed that HDAC inhibitors such as vorinostat enhance viral gene expression by disrupting H3K9me3-mediated repression, while EZH2 blockade reduces H3K27me3 deposition and destabilizes latency. BET inhibitors (e.g., JQ1) interfere with bromodomain binding to acetylated histones, preventing reactivation of lytic promoters [43,44]. These findings underscore that HSV latency is highly dependent on chromatin remodeling and suggest that epigenetic drugs may serve as precision tools to either awaken or silence latent reservoirs.

**Figure 1:**
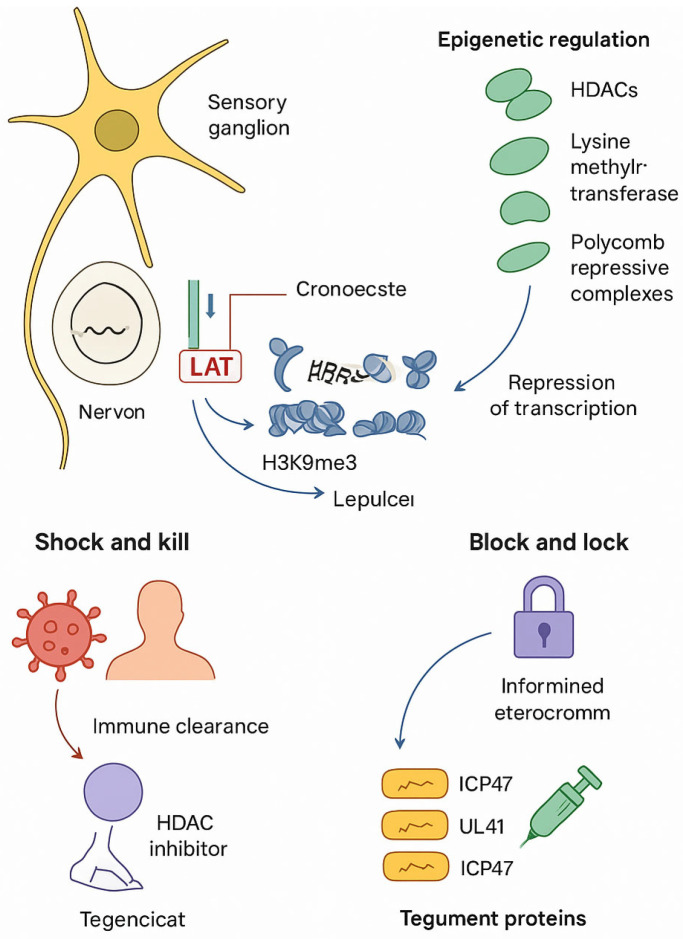
Molecular basis of HSV latency and therapeutic target.

In addition, tegument proteins, such as VP16 and ICP0, represent direct targets for preventing reactivation, while immunomodulatory approaches (therapeutic vaccines, cytokines, checkpoint inhibitors) aim to restore antiviral immunity. At the molecular level, therapeutic vaccines based on glycoprotein D and multivalent subunits enhance neutralizing antibody titers and CD4+/CD8+ T cell responses. Interferons (α/β) activate STAT1/STAT2 signaling, inducing antiviral ISGs that suppress HSV replication in mucosal tissues. Meanwhile, immune checkpoint inhibitors targeting the PD-1/PD-L1 axis reinvigorate exhausted HSV-specific T cells, restoring their cytolytic capacity against latently infected neurons. These immunotherapeutic approaches aim to rebalance the defective host–virus interaction characteristic of chronic HSV infection [45,46]. Balancing antiviral efficacy with host safety is critical for clinical translation (Figure 1) [47,48,49,50,51,52,53,54,55,56,57].

### 1.3. Current Topical and Systemic Therapeutic Strategies for HSV Infections

HSV infections are primarily treated with nucleoside analogues inhibiting viral DNA polymerase [58,59,60,61,62,63,64]. The choice of systemic versus topical therapy depends on disease severity, clinical presentation, and recurrence frequency [65,66,67,68,69].

#### 1.3.1. Systemic Antiviral Therapies

Systemic antivirals are indicated for severe, disseminated, or recurrent infections. Key agents include the following:

- **Acyclovir:** Guanine analogue activated by viral thymidine kinase, reducing symptom duration and viral shedding [70,71,72,73,74,75,76].
- **Valacyclovir:** Prodrug of acyclovir with improved oral bioavailability, used in episodic and suppressive therapy [77,78,79,80,81,82,83].
- **Famciclovir:** Prodrug of penciclovir, alternative in patients intolerant or resistant to acyclovir [84]. Novel systemic agents include helicase–primase inhibitors (e.g., pritelivir) and broad-spectrum antivirals targeting viral fusion or DNA packaging [85,86]. Systemic therapy can be episodic or suppressive, tailored to recurrence frequency and patient risk factors [87,88,89].

#### 1.3.2. Topical Antivirals

Topical antivirals are mainly used for mild-to-moderate orolabial HSV-1 infections. Agents include the following:

- **Acyclovir 5% cream/ointment** [90].
- **Penciclovir 1% cream** [91,92,93,94].
- **Docosanol 10% cream** [51,95].

Emerging formulations include helicase–primase inhibitor creams and siRNA-based therapies. Effectiveness depends on early application, and topical therapy is rarely indicated for genital HSV except when systemic options are contraindicated.

#### 1.3.3. Limitations of Conventional Therapies

Both systemic and topical therapies face limitations: inability to eradicate latent virus, reduced efficacy in immunocompromised hosts, potential drug resistance, and persistent asymptomatic shedding [96,97]. These challenges motivate the development of novel agents and combination approaches targeting multiple mechanisms, including latency and immune modulation [93,98,99].

### 1.4. Combination and Emerging Therapies

Alongside systemic and topical antivirals, recent RCTs have highlighted the clinical potential of light-based therapies. Both photobiomodulation therapy (PBMT) alone and PBMT in combination with antimicrobial photodynamic therapy significantly improved healing time and symptom relief in recurrent herpes labialis. These results position PBMT and PDT as promising adjunctive modalities under investigation [100,101,102,103]. Combination strategies may include traditional antivirals with epigenetic modulators; therapeutic vaccines with immune checkpoint inhibitors; or gene-editing tools, followed by immunostimulation [104,105,106,107,108,109,110,111,112,113,114,115,116,117,118]. While many approaches remain preclinical or in early clinical trials, they offer promise for long-term suppression or potential eradication of HSV [119,120,121,122,123].

## 2. Materials and Methods

### 2.1. Methodology

**Figure 2:**
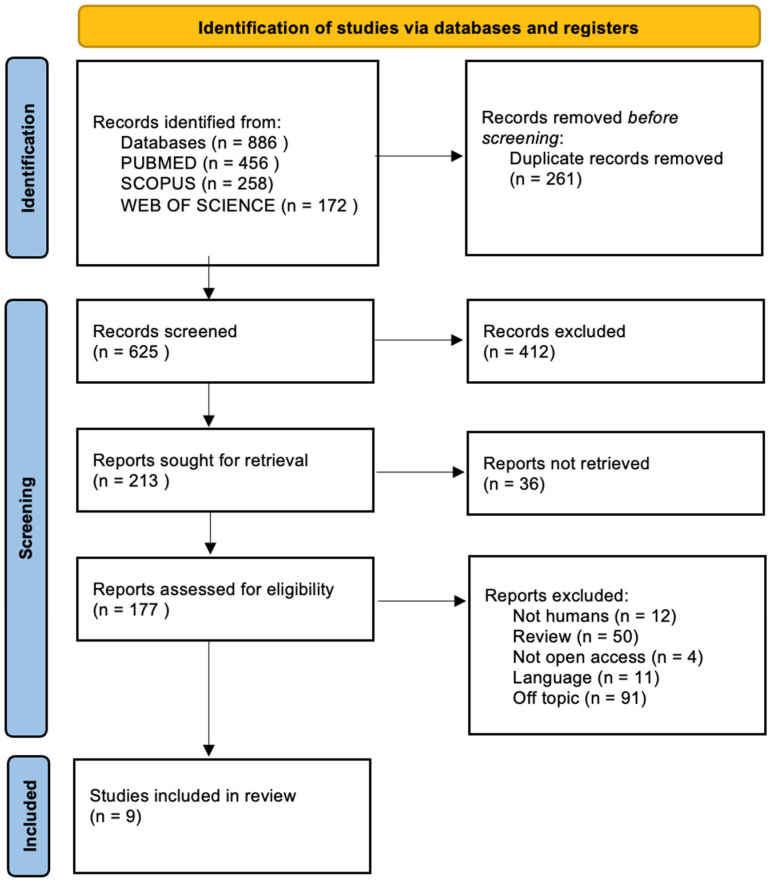
PRISMA flow diagram.

This systematic review was conducted following the PRISMA (Preferred Reporting Items for Systematic Reviews and Meta-Analyses) guidelines to ensure transparency and methodological rigor throughout the research process. This systematic review is currently being assessed for registration and has been issued the ID CDR 1106666. The PRISMA checklist was used to guide each phase of the review, including the development of the search strategy, selection of studies, data extraction, and assessment of methodological quality, thereby minimizing the risk of bias and enhancing reproducibility (Figure 2).

### 2.2. Literature Search

A comprehensive electronic search was performed in PubMed, Scopus, and Web of Science to identify relevant articles published between January 2005 and August 2025. The search strategy combined controlled vocabulary and free-text terms related to Herpes Simplex Virus (HSV) infection and treatment, including “oral herpes simplex virus,” “HSV-1 infection,” “herpes labialis,” “topical therapy,” “systemic therapy,” “antiviral agents,” “acyclovir,” “valacyclovir,” “penciclovir,” “resistance,” and “recurrent herpes.” Boolean operators (AND, OR) were used to maximize sensitivity and specificity. For example, combinations such as “oral herpes simplex virus” AND “topical therapy” OR “systemic therapy” were applied to capture studies addressing either localized or systemic interventions.

The search was restricted to articles published in English and available in full text. Due to practical limitations, only open-access publications were considered. While this approach ensured full-text accessibility and transparency, it may have introduced potential publication and language bias.

### 2.3. Eligibility Criteria

Study selection was based on the PICOS framework:

Population (P): Individuals of any age diagnosed with HSV-1 or HSV-2 infection.

Intervention (I): Topical or systemic therapeutic approaches, including antiviral drugs (e.g., acyclovir, valacyclovir, famciclovir), immunomodulatory agents, and novel topical formulations.

Comparison (C): Placebo, no treatment, or alternative therapeutic regimens (e.g., different dosing schedules or administration routes).

Outcome (O): Clinical efficacy in terms of lesion duration, symptom severity, frequency of recurrence, viral shedding, or patient-reported outcomes.

Study design (S): Randomized controlled trials (RCTs), cohort studies, case-control studies, and prospective observational studies.

Only original research articles reporting validated clinical outcomes were included to ensure scientific robustness and clinical relevance.

### 2.4. Exclusion Criteria

The following publications were excluded:

Animal or in vitro studies, to maintain clinical applicability.

Review articles (narrative, systematic, or meta-analyses), to avoid duplication of secondary evidence.

Studies not reporting therapeutic outcomes related to HSV management.

Articles not published in English or not available in full text. This exclusion strategy was applied to ensure inclusion of high-quality, clinically meaningful primary studies aligned with the review objectives.

Figure 2 shows the flow diagram illustrating the study selection process according to the PRISMA guidelines. The diagram shows the number of records identified, screened, assessed for eligibility, and included in the final review, along with reasons for exclusions at each stage.

## 3. Results and Discussion

The electronic search of the three databases identified a total of 886 studies: specifically, 456 on PubMed, 172 on Web of Science, and 258 on Scopus. A total of 261 duplicates were identified and removed. After deduplication, all titles and abstracts were screened from 625 articles. Of these, 412 were excluded after checking the relevance of the topic by title and abstract. Finally, 168 studies were excluded for the following reasons:

- Not involving humans (*n* = 12);
- Review (*n* = 50);
- Not available in open access (*n* = 4);
- Language not in English (*n* = 11);
- Off-topic (*n* = 91).

Finally, nine articles were selected. The selection process is summarized in Figure 2, and the articles enrolled for the discussion section are summarized in Table 1.

### 3.1. Risk of Bias Assessment

The methodological quality of the nine included clinical trials was assessed using the Cochrane Risk of Bias 2.0 tool. Overall, most studies demonstrated a low risk of bias across key domains, particularly in randomization and outcome measurement.

Some studies were rated as having “some concerns” (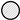), reflecting methodological limitations, such as missing outcome data, patient-reported outcomes (PROs) without blinding, or potential selective reporting. These factors do not compromise the overall validity of the study but suggest that results should be interpreted with appropriate caution.

The retrospective case series by Heidenreich et al. (2020) [131] was rated as high risk of bias (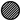) due to its non-randomized design and absence of a control group. This substantially reduces the internal validity of the study, and the evidence from this work should be considered lower quality compared to the randomized trials.

Table 2 presents a visual summary of domain-specific risk-of-bias assessments using a traffic light plot, with an updated legend clarifying the interpretation of the color codes.

Legend (RoB 2.0 Color Code).

- 🟢 Low Risk of Bias—Methodology is robust with minimal risk of bias.
- 🟡 Some Concerns—One or more domains present methodological limitations (e.g., missing data, self-reported outcomes without blinding, selective reporting). These aspects may introduce bias but do not invalidate the study; results should be interpreted with caution.
- 🔴 High Risk of Bias—Substantial methodological flaws (e.g., lack of randomization, absence of a control group) that significantly compromise the overall validity of the study.

### 3.2. Topical Therapies

Topical antiviral treatments, including acyclovir, docosanol, and newer formulations, are primarily indicated for local symptom control in Herpes Simplex Labialis (HSL). These agents act at the site of application to reduce lesion duration and alleviate discomfort, particularly when applied early in the course of an outbreak. Limited systemic absorption restricts their effect on recurrence prevention or systemic viral activity [126,129]. Several innovative strategies have been explored to enhance the local efficacy of topical therapies. These include combination formulations, such as acyclovir–hydrocortisone creams, and alternative delivery methods, including photodynamic therapy and advanced topical vehicles. Such approaches aim to improve symptom control and lesion resolution without altering systemic management. Topical treatments are particularly useful in mild or localized episodes, during initial outbreaks, or in patients for whom systemic therapy is contraindicated [127].

Formulation science plays a critical role in optimizing topical therapy, including improvements in penetration, retention, and patient adherence [124,125]. Adjunctive technologies, such as phototherapy, are also being evaluated for their potential to enhance local antiviral activity [130,132]. In immunocompromised patients or in cases of antiviral resistance, topical cidofovir or foscarnet may provide localized control with reduced systemic exposure.

### 3.3. Systemic Therapies

Systemic antiviral agents, including acyclovir, valacyclovir, and famciclovir, act throughout the body to target both active lesions and viral replication. They are indicated for episodic treatment of moderate to severe HSL and for suppressive therapy in patients with frequent or high-risk recurrences. Evidence from randomized trials supports the use of short-course, high-dose regimens that aim to achieve rapid symptom resolution and adherence-friendly treatment schedules.

#### 3.3.1. Longitudinal Contributions by Spruance et al.

Spruance et al. (2002–2006) provided substantial evidence regarding systemic therapy through a series of multicenter, double-blind, placebo-controlled trials. High-dose regimens of valacyclovir and famciclovir were studied, showing consistent benefits in healing time and symptom management. Single-dose famciclovir regimens were also evaluated, highlighting potential adherence advantages while maintaining clinical effectiveness. These studies provide a methodologically robust foundation for the use of systemic therapy in episodic and suppressive treatment of HSL [125,126].

#### 3.3.2. Non-Specific Vaccine Effects

Therapeutic strategies targeting host immune modulation are emerging as adjuncts to systemic therapy [133]. Vaccine-based approaches and cytokine-mediated immunostimulation may enhance innate and adaptive immune responses beyond the target pathogen. Evidence includes case reports of rapid recovery in severe HSV and herpes zoster episodes potentially linked to non-specific immunostimulatory effects. Live attenuated vaccines have shown potential to activate broader immune pathways, which could contribute to long-term management of HSV infections. Integration of such immune-modulatory approaches with systemic antiviral therapy may offer novel avenues to reduce recurrence and improve clinical outcomes.

### 3.4. Final Considerations

Recent literature emphasizes early intervention, drug bioavailability, and the potential benefits of combining antiviral and anti-inflammatory mechanisms. Systemic therapies are central for managing symptoms and preventing recurrences, while topical therapies serve as supportive options for localized disease. Novel delivery systems, immunomodulatory strategies, and formulation advances may further enhance therapeutic potential.

Systemic regimens, including daily suppressive valacyclovir or short-term prophylactic schedules, have demonstrated applicability across different clinical scenarios, including procedure-induced reactivation and frequent outbreaks. Topical agents remain relevant for symptom management, particularly when enhanced by optimized formulations or adjunctive technologies [127,128].

Future research should integrate traditional clinical endpoints with patient-centered outcomes, including quality of life, recurrence frequency, and functional impact. This approach will support more personalized, mechanism-based treatment strategies for both topical and systemic management of oral HSV infections.

## 4. Conclusions

This systematic review highlights the role of systemic and topical antiviral therapies for Herpes Simplex Virus (HSV) infections, focusing on mechanisms of action and recent innovations. Systemic agents, such as valacyclovir and famciclovir, provide robust efficacy in lesion management, recurrence control, and viral suppression, supporting both episodic treatment and prophylactic use in patients with frequent recurrences or known triggers, such as dental procedures.

Topical therapies serve as adjunctive options, particularly in initial episodes or when systemic therapy is contraindicated. Advances in topical delivery systems; combination formulations (e.g., acyclovir with hydrocortisone); and emerging technologies, such as photobiomodulation and photodynamic therapy, may improve local symptom control and patient comfort, though further research is needed to validate these approaches.

Overall, early intervention, appropriate modality selection, and patient-specific considerations should guide treatment strategies. Future studies should focus on modality-specific outcomes, development of novel therapeutic approaches, integration of quality-of-life measures, and personalization of therapy according to patient-specific factors, including recurrence frequency, immune competence, and known triggers, to optimize outcomes and long-term management of HSV infections.

## Figures and Tables

**Table 1:** Summary of the included articles.

Author and Year	Study Type	Materials and Methods	Results
Sacks et al., 2001 [124]	Double-Blind, Placebo-Controlled Randomized Clinical Trial	737 immunocompetent adults randomized at 21 U.S. sites; treated at prodrome/erythema phase; docosanol 10% cream vs. placebo (PEG base); applied 5x daily; followed with twice-daily clinical visits.	Median healing time reduced by 18 h in docosanol group (*p* = 0.008); reduced symptom duration (*p* = 0.002), classic lesion healing time (*p* = 0.023), and ulcer stage duration (*p* < 0.001); mild AEs.
Spruance et al., 2002 [125]	Randomized, Double-Blind, Vehicle-Controlled Clinical Trials	Two parallel multicenter trials (ZOVA3003 and ZOVA3004); 2079 adults with recurrent herpes labialis randomized to 5% ACV cream or vehicle. Applied 5×/day for 4 days.	ACV reduced episode duration by 0.5–0.6 days (*p* < 0.01) and pain by 0.3–0.4 days (*p* < 0.02). Efficacy independent of lesion stage at treatment initiation.
Spruance et al., 2003 [126]	Multicenter, Randomized, Double-Blind, Placebo-Controlled Trials	Subjects ≥ 12 years with ≥3 herpes labialis episodes/year. Treated at prodrome with valacyclovir 2 g BID (1- or 2-day regimen) or placebo. Self-assessment diaries; clinician evaluations.	Both valacyclovir regimens significantly reduced episode duration (1.1–1.3 days), healing time, and pain. No added benefit from 2-day regimen. Prevention of lesion progression improved; safety comparable to placebo.
Baker & Eisen, 2003 [127]	Two Randomized, Double-Blind, Placebo-Controlled Trials	Oral valacyclovir 500 mg once daily vs. placebo for 16 weeks; *n* = 49 per group; participants with ≥4 recurrences/year; data from both studies pooled for analysis.	60% recurrence-free in valacyclovir group vs. 38% in placebo (*p* = 0.041); time to first recurrence longer with valacyclovir (13.1 vs. 9.6 weeks; *p* = 0.016); fewer recurrences (24 vs. 41); fewer adverse events in valacyclovir group (33% vs. 39%).
Miller et al., 2004 [128]	Randomized, Double-Blind, Placebo-Controlled clinical Trial	125 HSV-1 seropositive adults with ≥1 recurrence/year; valacyclovir 2 g twice on day of procedure, 1 g twice the next day; placebo group as control; outcomes assessed by clinical exam, viral culture, and PCR analysis of saliva over 1-week post-treatment.	Fewer clinical lesions in valacyclovir group (11.3% vs. 20.6%); reduced HSV-positive cultures (1.6% vs. 7.9%) and saliva PCR (4.0% vs. 7.9%); fewer recurrences/shedding at 72 hrs (11.3% vs. 27%, *p* = 0.026); shorter pain duration (3.2 vs. 6.2 days, *p* = 0.006).
Spruance et al., 2006 [129]	Multinational, Randomized, Double-Blind, Placebo-Controlled Trial	701 patients randomized; received famciclovir 1500 mg once, 750 mg twice/day for 1 day, or placebo; therapy self-initiated within 1 h of prodromal symptoms; healing assessed via diaries and visits.	Median healing times: 4.4 days (1500 mg), 4.0 days (750 mg × 2), 6.2 days (placebo). Famciclovir accelerated healing and symptom resolution vs. placebo. No significant difference between active groups. Mild AEs.
Hull et al., 2011 [130]	Randomized, Double-Blind Clinical Trial	2437 HSL patients randomized to self-initiate treatment with ME-609 (5% acyclovir + 1% hydrocortisone), acyclovir, or placebo; cream applied 5×/day for 5 days.	ME-609 prevented ulcerative lesions in 42% vs. 35% (acyclovir) and 26% (placebo); reduced cumulative lesion area by 50% vs. placebo. Healing time and tenderness also improved.
Heidenreich et al., 2020 [131]	Retrospective Observational Case Series	214 HCT patients screened for ACVr HSV-1 after failed high-dose acyclovir; treated with topical cidofovir/foscarnet or IV foscarnet.	6 patients developed ACVr HSV-1 stomatitis; remission achieved in 5 with topical or IV antivirals; 5/6 died from AML relapse.
Al-Hallak et al., 2024 [132]	Randomized Clinical Trial	60 participants randomized into 3 groups: (1) 5% Acyclovir + inactive laser, (2) PBMT with LLLT + placebo cream, (3) aPDT with 0.01% methylene blue + PBMT. Laser at 650 nm, 100 mW, 120 s/point. Pain measured at t0–t4; healing by crust detachment.	aPDT + PBMT significantly reduced pain at t1 (*p* = 0.011), t2 (*p* = 0.041), t3 (*p* = 0.005) vs. control and at t3 vs. PBMT (*p* = 0.020). Healing time shorter in aPDT + PBMT (3.2 ± 1.06 days) vs. PBMT (4.05 ± 1.32) and control (4.75 ± 1.25), *p* = 0.001.

**Table 2:** Risk of bias summary (traffic light plot).

Study (Author, Year)	Randomization Process	Deviations from Intended Interventions	Missing Outcome Data	Measurement of Outcome	Selection of Reported Result	Overall Risk of Bias
Sacks et al., 2001 [124]	🟢	🟢	🟢	🟢	🟢	🟢
Spruance et al., 2002 [125]	🟢	🟢	🟡	🟢	🟢	🟡
Spruance et al., 2003 [126]	🟢	🟢	🟢	🟡	🟢	🟡
Baker & Eisen, 2003 [127]	🟢	🟢	🟡	🟢	🟢	🟡
Miller et al., 2004 [128]	🟢	🟢	🟢	🟢	🟢	🟢
Spruance et al., 2006 [129]	🟢	🟢	🟢	🟡	🟢	🟡
Hull et al., 2011 [130]	🟢	🟢	🟡	🟢	🟢	🟡
Heidenreich et al., 2020 [131]	🔴	🔴	🔴	🔴	🔴	🔴
Al-Hallak et al., 2024 [132]	🟢	🟢	🟡	🟢	🟢	🟡

## Abbreviations

The following abbreviations are used in this manuscript:

ACV	Acyclovir
ACVr	Acyclovir-Resistant
AE	Adverse Event
AML	Acute Myeloid Leukemia
aPDT	Antimicrobial Photodynamic Therapy
ATP	Adenosine Triphosphate
AUC	Area Under the Curve
BID	Bis in die (twice daily)
CD4+	Cluster of Differentiation 4 (Helper T cells)
CD4+/CD8+	Cluster of Differentiation 4 and 8 (types of T lymphocytes)
CI	Confidence Interval
°C	Degrees Celsius
DMSO	Dimethyl Sulfoxide
DNA	Deoxyribonucleic Acid
DOAJ	Directory of Open Access Journals
EMEM	Eagle’s Minimum Essential Medium
FBS	Fetal Bovine Serum
FDA	Food and Drug Administration
g	Gram
HCl	Hydrochloric Acid
HCT	Hematopoietic Cell Transplantation
HIV	Human Immunodeficiency Virus
HSL	Herpes Simplex Labialis
HSV	Herpes Simplex Virus
HSV-1	Herpes Simplex Virus Type 1
HSV-2	Herpes Simplex Virus Type 2
IRB	Institutional Review Board
ITT	Intent-to-Treat
IV	Intravenous
LD	Linear Dichroism
LLLT	Low-Level Laser Therapy
MDPI	Multidisciplinary Digital Publishing Institute
ME-609	Combination of 5% Acyclovir and 1% Hydrocortisone
N	Normal (refers to concentration, e.g., 1 N HCl = 1 mol/L solution)
OTC	Over-the-Counter
P	Probability Value (used in significance testing)
PBMT	Photobiomodulation Therapy
PBS	Phosphate-Buffered Saline
PEG	Polyethylene Glycol
PFU	Plaque Forming Units
RCT	Randomized Clinical Trial / Randomized Controlled Trial
RHL	Recurrent Herpes Labialis
RNA	Ribonucleic Acid
ROS	Reactive Oxygen Species
SD	Standard Deviation
Th1	T Helper Type 1 (Proinflammatory Cytokine Response)
TLA	Three Letter Acronym
U.S.	United States
VAS	Visual Analog Scale
WHO	World Health Organization
wt/wt	Weight per weight
ZOVA3003/3004	Identifiers for the two clinical trial protocols
μg	Microgram
μL	Microliter
